# Supplementary material for: Microspheres from light—a sustainable materials platform
Source: Nat Commun. 2022 Sep 1;13:5132. doi: 10.1038/s41467-022-32429-3 (PMC9434521; doi:10.1038/s41467-022-32429-3)
Supplement: Supplementary file 1 — Supplementary Information [file 41467_2022_32429_MOESM1_ESM.pdf]

Microspheres from Light – A Sustainable Materials Platform – Supplementary Information

Laura Delafresnaye,[1,2,‡*] Florian Feist,[3,‡] Jordan P. Hooker[1,2] and Christopher Barner-Kowollik[1,2,*]

[1]School of Chemistry and Physics, Queensland University of Technology (QUT), Brisbane, QLD, Australia. [2]Centre for Materials Science, Queensland University of Technology (QUT), Brisbane, QLD, Australia. [3]Institute of Nanotechnology (INT), Karlsruhe Institute of Technology (KIT), Hermann-von-Helmholtz-Platz 1, 76344 Eggenstein-Leopoldshafen, Germany.
‡ These authors contributed equally.

Correspondence: laura.delafresnaye@qut.edu.au, christopher.barnerkowollik@qut.edu.au

1. Materials 2
2. Instrumentation 2
3. Monomer Synthesis 4
3.1. Synthesis of 4-hydroxy-2,5-dimethylisophthalaldehyde 4
3.2. Synthesis of Monomer AA1 (4-Methoxy-2,5-dimethylisophthalaldehyde) 4
3.3. Synthesis of Monomer AA2 (4-(2-(2-(2-methoxyethoxy)ethoxy)ethoxy)-2,5-dimethylisophthalaldehyde) 5
3.4. Synthesis of Monomer AA3 (4,4'-((1,4-phenylenebis(methylene)) bis(oxy))bis(2,5-dimethylisophthalaldehyde)) 5
3.5. Synthesis of monomer AA4 (4,6-Dimethoxy-2,5-dimethylisophthalaldehyde) 6
4. Particle Synthesis 7
5. SEC Characterization 10
6. Stability 11
6.1. Stability in solvent 11
6.2. DSC 14
6.3. TGA 14
7. Functionalization 15
7.1. Functionalization with tetrazole 15
7.2. Functionalization with PEG-thiol 16
8. NMR Spectra 17
9. LCMS Results 21
10. References 22

1. Materials

All materials were reagent grade and used as received, unless stated otherwise: 2,5-Dimethylphenol (≥99 %, Sigma-Aldrich), Formamidine acetate (TCI, >98%), Acetic anhydride (Acos Organics, +99%), , 2,5-Dimethylresorcinol (95 %, Sigma-Aldrich), Trifluoroacetic acid (99.9 % ABCR), Hexamethylene tetramine (99% ABCR), Methyl iodide (99 %, Merck), α,α-Dibromo-p-xylene (97 %, Sigma-Aldrich), Dioxane (VWR, *AnalaR Normapur*®, >99.5%), Potassium carbonate (99.9 %, Merck), 1,1'-(Methylenedi-4,1-phenylene)bismaleimide (95%, Sigma-Aldrich), 2,4-Toluene bismaleimide (Evonik), poly(ethylene glycol) methyl ether thiol (PEG-SH, M_n 2000 g mol^{-1}, Sigma-Aldrich), triethylamine (dry, ≥99.5%, Sigma-Aldrich), N,N-Dimethylformamide (DMF, anhydrous 99.8 %, Sigma-Aldrich), Acetonitrile (ACN, HPLC-grade, Fisher), Dimethyl sulfoxide (DMSO, anhydrous 99,9 %, Sigma-Aldrich), Methanol (MeOH, analytical reagent, Ajax Finechem), Tetrahydrofuran (THF, analytical reagent, Fisher), Chloroform (analytical reagent, Fisher), Cyclohexane (CH, analytical reagent, Ajax Finechem), Ethyl acetate (EA, analytical reagent, Fisher), Dichloromethane (DCM, analytical reagent, Fisher), 1,2,4-Trichlorobenzene (TCB, analytical reagent, Sigma-Aldrich), Acetonitrile-d_3 (99.8 %D, Cambridge Isotope Laboratories), Chloroform-*d* (99.8 %D, Cambridge Isotope Laboratories), Dimethylsulfoxide-d_6 (99.9 %D, Cambridge Isotope Laboratories).
4-(2-Phenyl-2H-tetrazol-5-yl)benzoic acid (Tetrazole 1) and 4-(2-(4-Methoxyphenyl)-2H-tetrazol-5-yl)benzoic acid (Tetrazole 2) were synthesized as per literature.[1]

2. Instrumentation

Nuclear Magnetic Resonance (NMR) Spectrometry: ^{1}H and ^{13}C-NMR spectra were recorded on a *Bruker* System 600 Ascend LH, equipped with a BBO-Probe (5 mm) with z-gradient (^{1}H: 600.13 MHz, ^{13}C 150.90 MHz) or on a Bruker AM 400 equipped with a PABBO-Probe (5 mm) (^{1}H: 400 MHz, ^{13}C: 101 MHz) The δ-scale was normalized relative to the solvent signal of $CHCl_3$ or DMSO for ^{1}H spectra and for ^{13}C spectra on the middle signal of $CDCl_3$ triplet, the DMSO quintet, or ACN septet. The multiplicities were reported using the following abbreviations: s for singlet, d for doublet, t for triplet, m for multiplet and br for broad signal.

Interchim XS420: Flash chromatography was performed on a Interchim XS420+ flash chromatography system consisting of a SP-in-line filter 20-µm, an UV-VIS detector (200-800 nm) The separations were performed using an Interchim dry load column and a Interchim Puriflash Silica HP 30 µm column after deposition on Celite® 565 (Sigma-Aldrich).

THF Size Exclusion Chromatography (SEC): The SEC measurements were conducted on a *PSS* SECurity[2] system consisting of a *PSS* SECurity Degasser, *PSS* SECurity TCC6000 Column Oven (35 °C), *PSS* SDV Column Set (8x150 mm 5 µm Precolumn, 8x300 mm 5 µm Analytical Columns, 100000 Å, 1000 Å and 100 Å) and an *Agilent* 1260 Infinity Isocratic Pump, *Agilent* 1260 Infinity Standard Autosampler, *Agilent* 1260 Infinity Diode Array and Multiple Wavelength Detector (A: 254 nm, B: 360 nm), *Agilent* 1260 Infinity Refractive Index Detector (35 °C). HPLC grade THF, stabilized with BHT, was used as eluent at a flow rate of 1 mL min^{-1}. Narrow disperse linear poly(styrene) (M_n: 266 g mol^{-1} to 2.52x10^{6} g mol^{-1}) and poly(methyl methacrylate) (M_n: 202 g mol^{-1} to 2.2x10^{6} g mol^{-1}) standards (*PSS* ReadyCal) were used as calibrants. All samples were passed over 0.22 µm PTFE membrane filters. Molecular weight and dispersity analyses were performed in *PSS* WinGPC UniChrom software (version 8.2).

Fluorescence Spectroscopy: The fluorescence intensities were measured using a Cary Eclipse Fluorescence Spectrophotometer from *Agilent Technologies*. Voltage was set to high voltage and the excitation wavelength was set to 390 nm with an excitation and emission slit of 5 nm (scan rate 600 nm min^{-1}). Samples were measured at ambient temperature in *Helma Analytics* quartz high precision cells with a path length of 10 mm.

LED and sunlight spectra were recorded with an Ocean Optics FLAME-T-UV-VIS spectrometer, sensitive from 200 to 850 nm (integration time of 10 ms).

Centrifuge: The particles were isolated by centrifugation using a mini centrifuge (Labco), at 15,000 rpm for 5 minutes (2 mL vial). The particles were isolated by centrifugation using a Sigma 3-16 L centrifuge at 5,000 rpm for 5 minutes (20 mL vial).

Scanning Electron Microscopy: SEM images were captured using a Tescan MIRA3 using an in-lens in beam SE detector at a 3 mm working distance using a 5 kV acceleration voltage (beam intensity = 8). Samples were prepared by dispersing the particles in ACN (except noted otherwise) and drop casting onto a silicon wafer glued onto a SEM stub using conductive carbon tape. Samples were coated with a 3 nm platinum layer. Analysis was conducted in ImageJ, with particle sizing and dispersity calculations based on counting a minimum of 250 particles, using the following equations:

$$D_n = \frac{\sum N_i D_i}{\sum N_i} \qquad (1)$$

$$D_w = \frac{\sum N_i D_i^4}{\sum N_i D_i^3} \qquad (2)$$

Where D_n is the number-average diameter, D_w is the weight-average diameter, N_i is the number of particles measured, and D_i is the diameter of the measured particle. The dispersity *Đ* can then be calculated as

$$Đ = \frac{D_w}{D_n} \qquad (3)$$

Liquid Chromatography coupled Mass Spectroscopy (LCMS): LCMS measurements were performed on an Agilent 1260 Infinity II system consisting of a quaternary pump (GB7111B;), autosampler (G7129A, 100 µL sample loop), a temperature-controlled column oven (G7114A) and a variable UV-VIS detector (G7114 A, VWD, flow cell G7114A 018, d = 10 mm, V = 14 µL). Separation was performed on a C18 HPLC-column (Agilent Poroshell 120 EC-C18 4,6x100mm, 2,7µm) operating at 40 °C. A gradient of ACN:H_2O 10:90 – 80:20 *v/v* (additive 10 mmol L^{-1} $NH_4CH_3CO_2$) at a flow rate of 1 mL·min^{-1} during 15 min was used as the eluting solvent. The flow was directed into an Agilent MSD (G6136BA, AP-ESI ion source). The instrument was calibrated in the m/z range 118-2121 in the positive mode and 113-2233 in the negative using a premixed calibration solution (Agilent). The following parameters were used: spray chamber flow: 12 L min^{-1}; drying gas temperature: 350 K, Capillary Voltage: 3000 V, Fragmentor Voltage: 100 V.

Differential Scanning Calorimetry (DSC) was conducted using a Netzsch DSC 204 F1 Phoenix. Samples (6 mg) were sealed into Netzsch Al sample capsules. 3 cycles of heating/cooling ramp at scanning speed of 10 °C min^{-1} were used over the temperature range from 0 °C to 150 °C with isothermal exposures (2 min) at 0°C and 150°C. A nitrogen sample purge flow of 20 mL min^{-1} was utilised. The data were analysed using TA Instruments Universal Analysis 2000 software (version 4.2E).

Thermogravimetric Analysis (TGA) was performed on a STA 449 F3 Jupiter from Netzsch. Samples (10 mg) were analysed in aluminum oxide pans at a heating rate of 20 °C min^{-1} from 30 °C to 800 °C under nitrogen atmosphere. The data were analysed using TA Instruments Universal Analysis 2000 software (version 4.2E).

3. Monomer Synthesis

3.1. Synthesis of 4-hydroxy-2,5-dimethylisophthalaldehyde

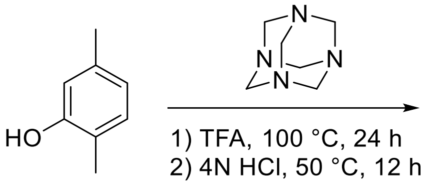

In a 250 mL round bottom flask, a solution of 2,5-dimethylphenol (5.00 g, 40.93 mmol, 1.00 eq) in 32.7 mL TFA was prepared and hexamethylenetetramine (20.80 g, 163.71 mmol, 4.00 eq) was added. The resulting viscous solution was stirred under inert atmosphere at 100 °C in an oil bath for 24 h. Subsequently, the reaction mixture was cooled to ambient temperature and 72 mL 4 N HCl were added. The mixture was heated to 50°C whilst passing through nitrogen for 12 h. Finally, the resulting solution was cooled at ambient temperature, diluted with 50 mL water and cooled in a refrigerator at 7 °C overnight. The resulting precipitate was filtered off, washed with 10 mL cold water and dried in vacuum. The resulting crude product was either purified *via* flash chromatography (EA:CH 10:90-20:80 *v/v*) or sublimated under reduced pressure at 60 °C. The product was obtained as a slightly yellow crystals (4.30 g, 59 % yield).

The NMR spectra are consistent with earlier reported results.[2,3]
^{1}H NMR (600 MHz, Chloroform-*d*) *δ*: 12.98 (s, 1H), 10.48 (s, 1H), 10.24 (s, 1H), 7.86 (s, 1H), 2.93 (s, 3H), 2.27 (s, 3H).
^{13}C NMR (151 MHz, Chloroform-*d*) *δ*: 195.58, 190.06, 166.38, 144.16, 140.00, 126.25, 126.12, 117.81, 15.05, 12.14.

3.2. Synthesis of Monomer AA1 (4-Methoxy-2,5-dimethylisophthalaldehyde)

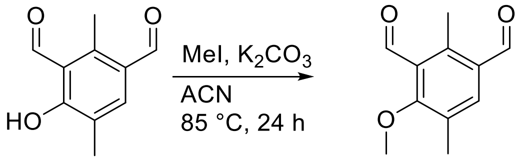

Monomer AA1

4-Hydroxy-2,5-dimethylisophthalaldehyde (3.00 g, 16.84 mmol, 1.00 eq) was dissolved in 100 mL dry acetonitrile under inert atmosphere. Methyl iodide (1.57 mL, 3.58 g, 1.50 eq) was added *via* syringe. Anhydrous K_2CO_3 (2.70 g, 21.05 mmol, 1.25 eq) was subsequently added and the suspension stirred at 85 °C for 24 h until complete consumption of the starting material. Afterwards, the reaction mixture was cooled to ambient temperature, 150 mL 0.1 N HCl and 250 mL ethyl acetate were added, the organic phase separated, the aqueous phase washed twice with 50 mL ethyl acetate and the combined organic phases washed with brine, and finally dried over $MgSO_4$. The volatiles were removed under reduced pressure and the residual crude product was purified *via* flash chromatography (isocratic cyclohexane: ethyl acetate 85:15 *v/v*). The product was obtained as colorless crystals (3.04 g, 94 % yield).

The NMR spectra are consistent with earlier reported results.[2,3]
^{1}H NMR (600 MHz, Chloroform-d) *δ*: 10.54 (s, 1H), 10.37 (s, 1H), 7.90 (s, 1H), 3.88 (s,3H), 2.83 (s, 3H), 2.35 (s, 3H).
^{13}C NMR (151 MHz, Chloroform-d) *δ*: 192.90, 190.86, 166.80, 142.19, 138.03, 131.25,130.19, 129.36, 62.99, 15.58, 14.25.

3.3. Synthesis of Monomer AA2 (4-(2-(2-(2-methoxyethoxy)ethoxy)ethoxy)-2,5-dimethylisophthalaldehyde)

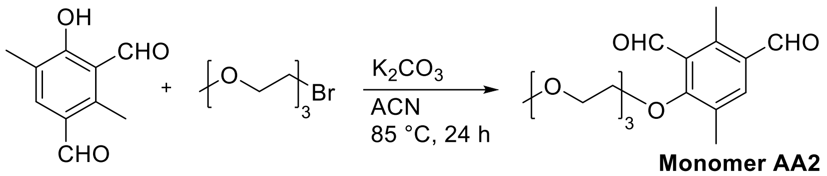

Monomer AA2

4-hydroxy-2,5-dimethylisophthalaldehyde (500 mg, 2.806 mmol, 1.00 eq) was dissolved in 17 mL dry acetonitrile under inert atmosphere. 1-bromo-2-(2-(2-methoxyethoxy)ethoxy)ethane (797 mg, 3.51 mmol, 1.25 eq) was dissolved in 5 mL dry acetonitrile and added via syringe. Anhydrous K_2CO_3 (414 mg, 3.22 mmol, 1.15 eq) was then added and the suspension stirred at 85 °C for 36 h until complete consumption of the starting material. Afterwards the reaction mixture was cooled to room temperature, 150 mL 0.1 N HCl and 250 mL ethyl acetate were added, the organic phase separated, the aqueous phase washed twice with 50 mL ethyl acetate and the combined organic phases washed with brine, and finally dried over $MgSO_4$.The volatiles were removed under reduced pressure and the residual crude product was purified *via* flash chromatography (gradient cyclohexane: ethyl acetate 85:15 – 20:80 v/v). The product was obtained as slightly yellowish oil (473 mg, 52 % yield).

^{1}H NMR (400 MHz, Acetonitrile-*d$_3$*) *δ*: 10.54 (s, 1H), 10.29 (s, 1H), 7.87 (s, 1H), 4.15 – 4.09 (m, 2H), 3.80 – 3.72 (m, 2H), 3.62 – 3.51 (m, 6H), 3.48 – 3.41 (m, 2H), 3.28 (s, 3H), 2.76 (s, 3H), 2.34 (s, 3H). refer to **Supplementary Figure 15**.
^{13}C NMR (101 MHz, Acetonitrile-*d$_3$*) *δ*: 194.30, 191.92, 165.75, 141.75, 138.18, 131.72, 130.83, 130.17, 75.24, 72.19, 70.90, 70.69, 70.65, 70.37, 58.46, 15.60, 14.01. Refer to **Supplementary Figure 16**.

LCMS: Refer to **Supplementary Figure 23**.

3.4. Synthesis of Monomer AA3 (4,4'-((1,4-phenylenebis(methylene)) bis(oxy))bis(2,5-dimethylisophthalaldehyde))

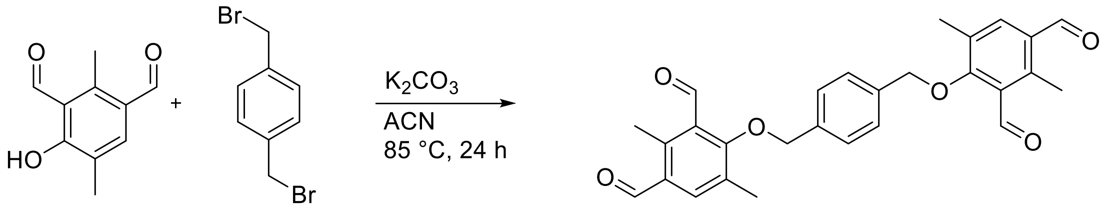

Monomer AA3

4-Hydroxy-2,5-dimethylisophthalaldehyde (200 mg, 1.12 mmol, 2.25 eq) was dissolved in 20 mL dry acetonitrile under inert atmosphere. α,α-Dibromo-p-xylene (131 mg, 0.50 mmol, 1.00 eq) was added. Anhydrous K_2CO_3 (2.70 g, 21.05 mmol, 1.25 eq) was then added and the suspension stirred at 85 °C for 24 h until complete consumption of the starting material. Afterwards, the reaction mixture was cooled to ambient temperature, 15 mL 0.1 N HCl and 250 mL DCM were added, the organic phase separated, the aqueous phase washed three times with 25 mL DCM and the combined organic phases washed with brine, and dried over $MgSO_4$. The volatiles were removed under reduced pressure and the residual crude product was purified *via* flash chromatography (gradient DCM:MeOH 99:1 – 95:5 *v/v*). The product was obtained as colorless solid (177.2 mg, 81 % yield).

^{1}H NMR (600 MHz, Chloroform-d) *δ*: 10.50 (s, 2H), 10.40 (s, 2H), 7.94 (s, 2H), 7.45 (s, 4H), 5.00 (s, 4H), 2.84 (s, 6H), 2.37 (t, J = 0.7 Hz, 6H). Refer to **Supplementary Figure 17**.

^{13}**C NMR** (151 MHz, Chloroform-d) *δ*: 192.79, 190.86, 165.12, 142.17, 138.03, 136.33, 131.48, 130.43, 129.74, 128.66, 77.31, 16.05, 14.30. Refer to **Supplementary Figure 18**.

3.5. Synthesis of monomer AA4 (4,6-Dimethoxy-2,5-dimethylisophthalaldehyde)

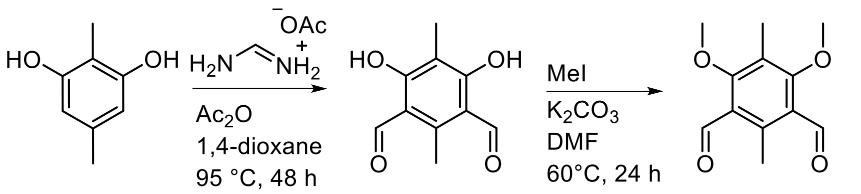

Monomer AA4

AA4 was synthesized from 2,5-dimethylresorcinol in a two-step procedure with an overall yield of 82 %.

4,6-dihydroxy-2,5-dimethylisophthalaldehyde was synthesized according to a modified literature procedure[4]:

Formamidine acetate (6.03 g, 57.94 mmol, 4.00 eq) and dioxane (70 mL) were added to a round bottom pressure flask (250 mL, 4.2 bar max.) and heated at 95 °C under vigorous stirring. Acetic anhydride (21.91 mL, 23.66 g, 231.77 mmol, 16.00 eq) was added once the target temperature had been reached and stirring was continued until the formamidine acetate fully dissolved (typically 30 min). At this point, the resorcin (2.00 g, 14.49 mmol, 1.00 eq) was added in one portion. The solution was degassed by passing through nitrogen for 15 min and the flask was subsequently sealed and heated for 48 h at 105 °C. After cooling to ambient temperature, the volatiles were removed under reduced pressure at a bath temperature of 50 °C, followed by the addition of aqueous 2 N hydrochloric acid and degassing the solution by passing through nitrogen for 15 min. The solution was heated under nitrogen atmosphere for 12 h, cooled to ambient temperatures and the beige precipitate filtered and washed with water (50 mL) and diethyl ether (50 mL). Afterwards the beige solid was dried under vacuum and used for the next step without further purification (2.45 g, 87 % yield).

^{1}**H NMR** (400 MHz, DMSO) *δ*: 13.40 (s, 2H), 10.30 (s, 2H), 2.85 (s, 3H), 1.97 (s, 3H). Refer to **Supplementary Figure 19**.
^{13}**C NMR** (101 MHz, DMSO) *δ*: 195.89, 166.77, 150.09, 112.64, 108.89, 79.18, 11.48, 6.38. Refer to **Supplementary Figure 20**.

4,6-dimethoxy-2,5-dimethylisophthalaldehyde
4,6-dihydroxy-2,5-dimethylisophthalaldehyde (1500 mg, 7.72 mmol, 1.00 eq) was dissolved in 46.5 mL dry DMF under inert atmosphere. Methyl iodide (1.44 mL, 3.29 g, 23.17, 3.00 eq) was added *via* syringe. Anhydrous K_2CO_3 (2.48 g, 19.31 mmol, 2.50 eq) was added and the suspension stirred at 60 °C for 16 h until complete consumption of the starting material. Afterwards, the reaction mixture was cooled to ambient temperature, 150 mL water and 150 mL ethyl acetate were added, the organic phase separated, and the aqueous phase washed twice with 50 mL ethyl acetate. The combined organic phases were washed with brine, dried over $MgSO_4$, the volatiles removed under reduced pressure and the residual crude product was recrystallized from *n*-hexane (50 mL) at -18 °C to obtain transparent crystal needles (1.61 g, 94 % yield).

^{1}**H NMR** (500 MHz, $CDCl_3$) *δ*: 10.47 (s, 2H), 3.86 (s, 6H), 2.74 (s, 3H), 2.25 (s, 3H). Refer to **Supplementary Figure 21**.
^{13}**C NMR** (126 MHz, $CDCl_3$) *δ*: 192.23, 167.26, 143.09, 126.20, 123.70, 63.12, 16.09, 8.91. Refer to **Supplementary Figure 22**.
LCMS: Refer to **Supplementary Figure 24**.

4. Particle Synthesis

For a typical reaction, stock solutions of AA and BB monomers were prepared in acetonitrile at a concentration of 5 mmol L^{-1}. Then, 1 mL of each solution was passed through a 2.5 µm PTFE syringe filter and placed in a crimp cap vial ($c_{AAmonomer} = c_{BBmonomer} = 2.5$ mmol L^{-1}; V = 2 mL). Oxygen was removed by passing through a stream of nitrogen (N_2) for 5 min. Under irradiation with a 3W or 10 W LED (λ = 360-390 nm, 2-4 cm distance, **Supplementary Figure 2**) on a ThermoFisher Scientific Bottle/Tube Roller at 10 rpm, the clear solution gradually becomes heterogeneous (**Supplementary Figure 1**A). After 4h, the turbid solution was centrifuged (15,000 rpm, 5 min), the supernatant was removed, and the solid pellet washed with THF twice. The resulting particles were redispersed in ACN and characterized via SEM. For larger scale, 10 mL of each solution was placed in a 20 mL crimp vial and the particles were centrifuged at 5,000 rpm for 5 min. For the sunlight experiments, the same conditions were used without the LED and by placing the bottle roller outside for 4-8h (**Supplementary Figure 1**B).
NB: AA3 was used as 1.25 mmol L^{-1} and BB as 2.5 mmol L^{-1} to have 0.5 eq. (4 reactive moieties) for 1.0 eq. of bismaleimide (2 reactive moieties).

The yield – which represents the conversion of monomer to particle – was calculated on the 20 mL batch to limit incertitude and was calculated as follows:

$$\text{Yield (\%)} = \frac{\text{weight}_{\text{dry particles}}}{\text{weight}_{\text{dry particles}} + \text{weight}_{\text{dry supernatant}}} \times 100 \quad (4)$$

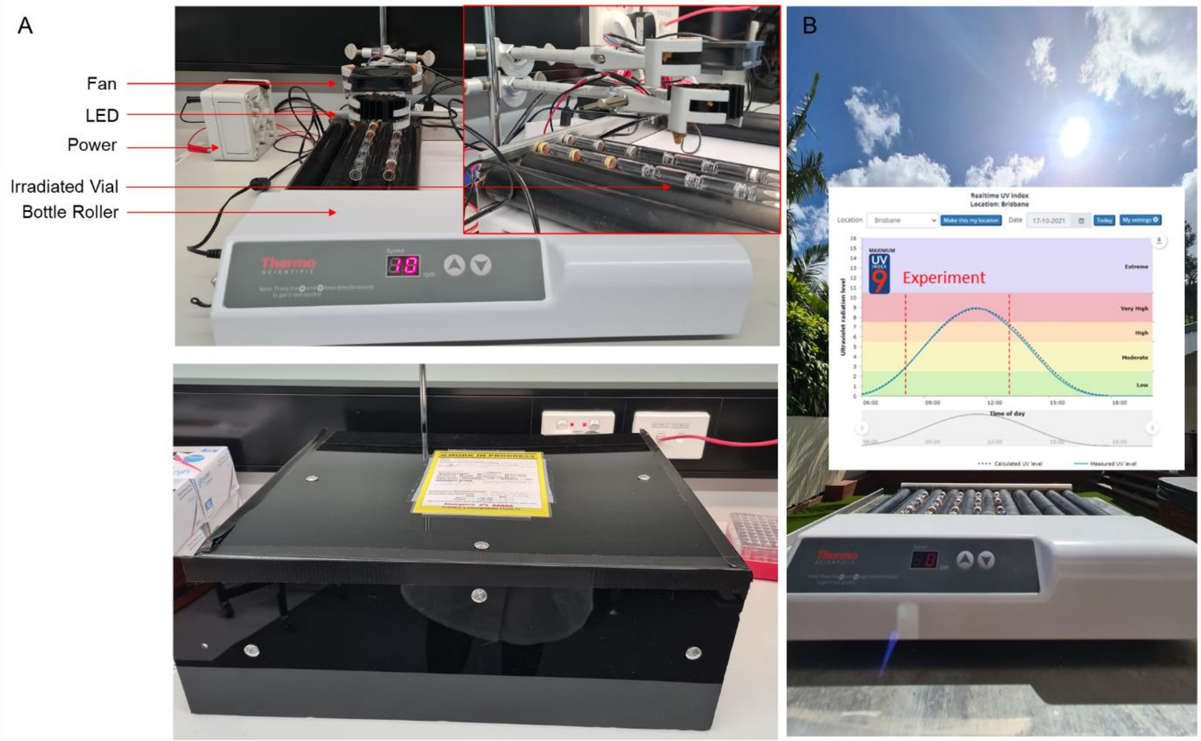

Supplementary Figure 1. Set-up for the particle synthesis. A Under 10W 365 nm LED UV light. **B** Outdoor in the sole presence of sun. Date 17th October 2021, 8h30-13h, Brisbane, Australia (Latitude: -27.530620; longitude: 153.059640). Daily exposure on that day 26.9 MJ m^{-2}.[5] The graph shows the UV data collected by the Australian Radiation Protection and nuclear Safety Agency and is available online.[6]

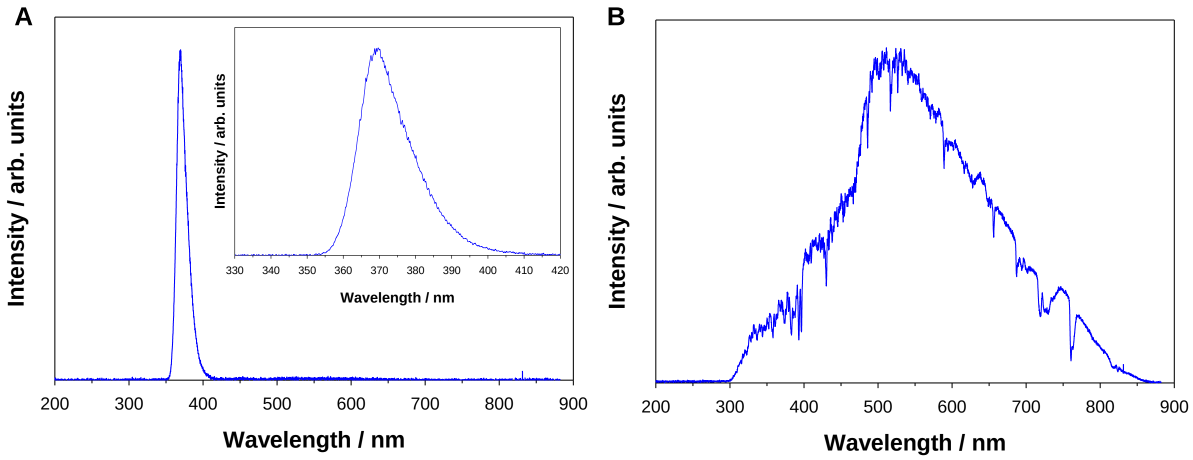

Supplementary Figure 2. **Emission spectra used for photoreactions. A** 3 W LED centred at 365 nm. **B** Sunlight.

Supplementary Table 1. **Particles counting (SEM) and reactions conditions of the particles' synthesis.**

Run	Monomer AA	Monomer BB	Reactions Conditions	Daily exposure (MJ m^2)	D_n (µm)	*Đ*
1.1_A	1	1	3 W, 4 cm	N/A	0.79	1.15
1.1_B	1	1	10 W 365 nm	N/A	1.06	1.03
1.1_C	1	1	sunlight	26.9	0.76	1.12
1.1_D	1	1	10 W 365 nm	N/A	1.50	1.07
1.2	1	2	sunlight	24.6	0.94	1.17
2.1	2	1	sunlight	18.0	0.69	1.11
2.2	2	2	sunlight	18.0	1.85	1.15
3.1	3	1	sunlight	18.0	0.94	1.02
3.2	3	2	sunlight	18.0	0.44	1.04
4.1	4	1	sunlight	22.1	0.88	1.01
4.2	4	2	sunlight	22.1	0.92	1.03

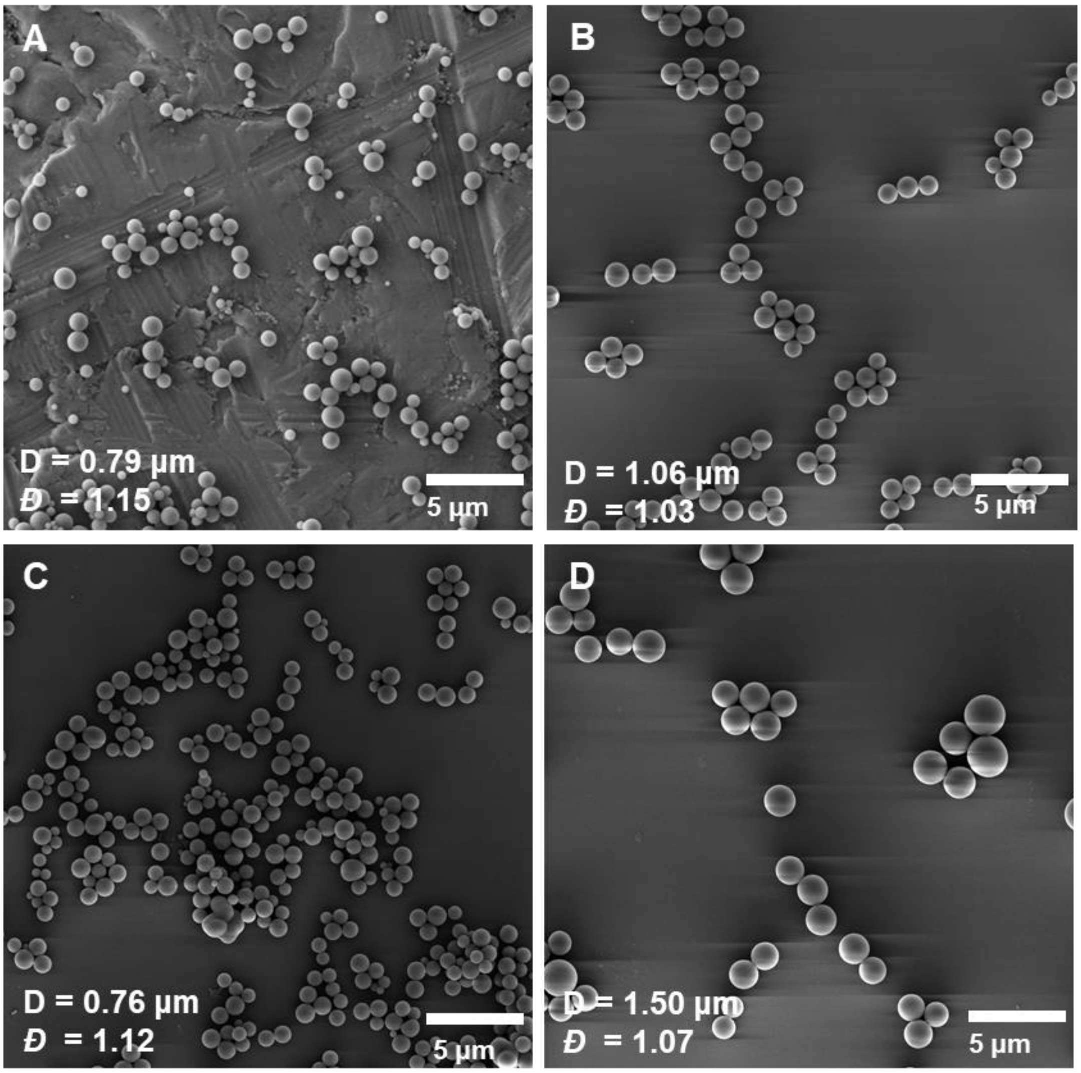

Supplementary Figure 3. **SEM images of AA1/BB1 particles.** The particles are produced with a 3W 365 nm LED (**A**, run 1.1_A), 10 W 365 nm LED (**B**, run 1.1_B, **D**, run 1.1_D), and under Australian sunlight (**C**, run 1.1_C).

5. SEC Characterization

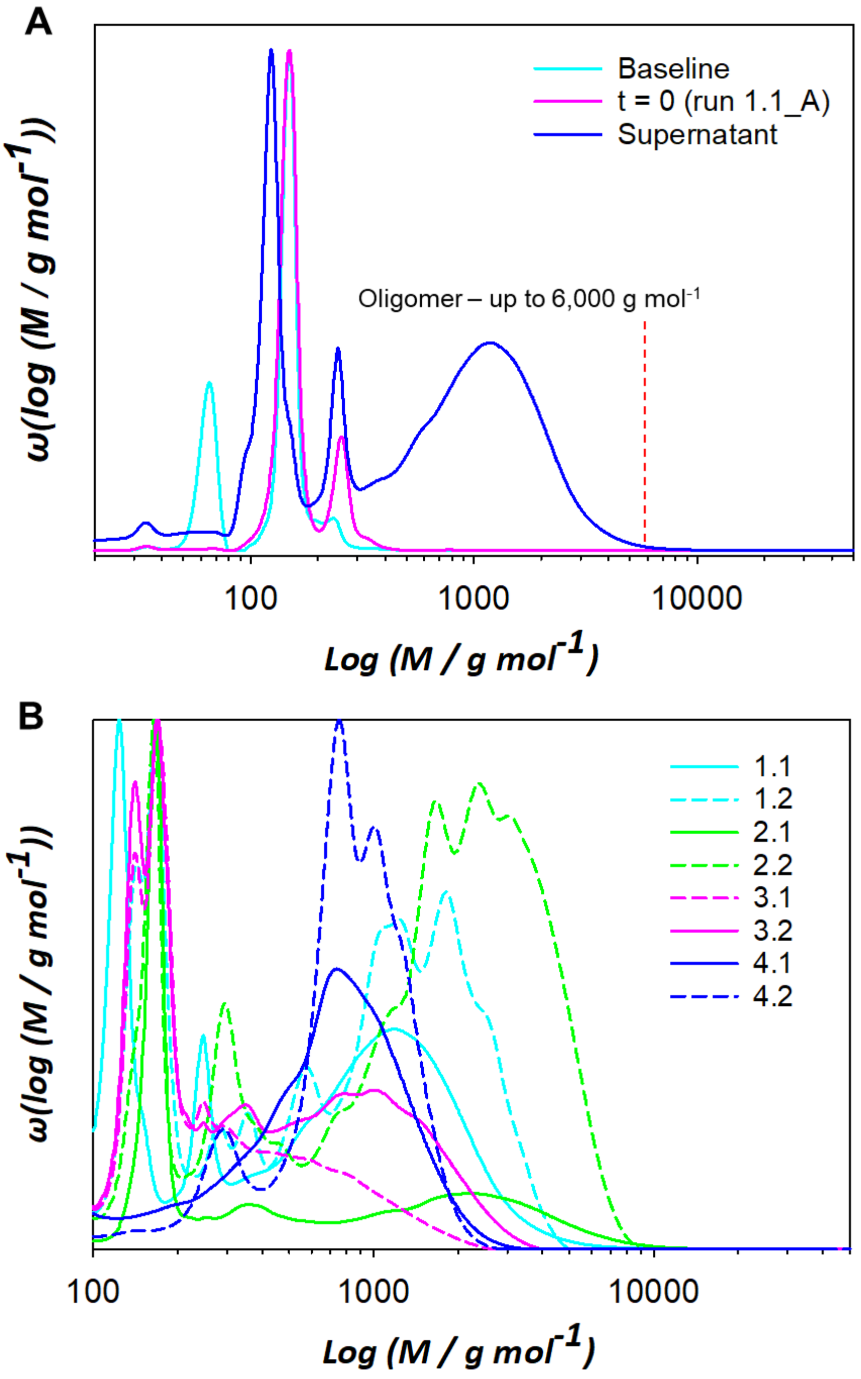

Supplementary Figure 4. **Size Exclusion Chromatograms (SEC) of supernatants. A** SEC of the initial mixture and supernatant of AA1/BB1 (run 1.1_A, PS calibration). Baseline (blank of pure THF) shows that small molecules are present, and we cannot conclude if there are residual AA and BB monomers (i.e 192 and 282 g mol^{-1}, respectively). **B**. SEC of the supernatant of run 1.1_A, 1.2, 2.1, 2.2, 3.1, 3.2, 4.1 and 4.2 (PS calibration).

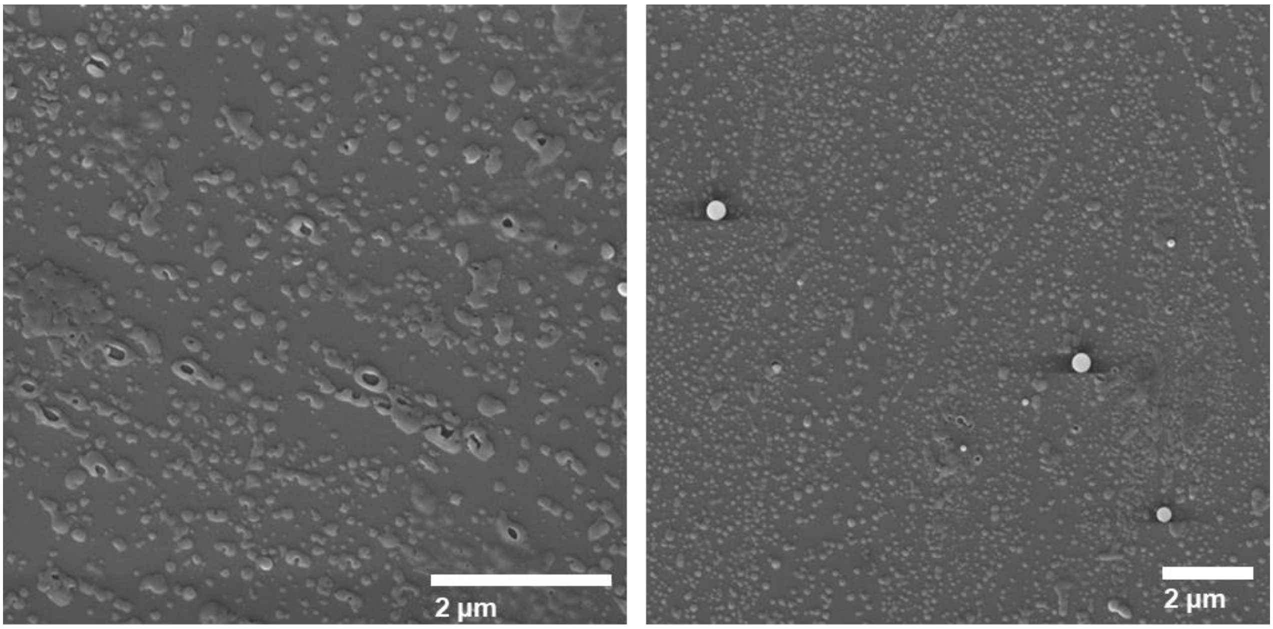

Supplementary Figure 5. **SEM images of supernatant.** SEM images of the supernatant of AA1/BB1 (run 1.1_A).

6. Stability

6.1. Stability in solvent

Particles AA1/BB1 (run 1.1_B) were synthesized as per standard procedures and washed 2 times with THF. The isolated particles were then redispersed in 2 mL of either ACN, THF, or Chloroform. A SEM was taken and noted as time 0. The solutions were stored at room temperature for 6 months and a SEM from the same vial was recorded every month. Prior to SEM, the solution was sonicated for 30 sec to ensure a good dispersion for SEM.

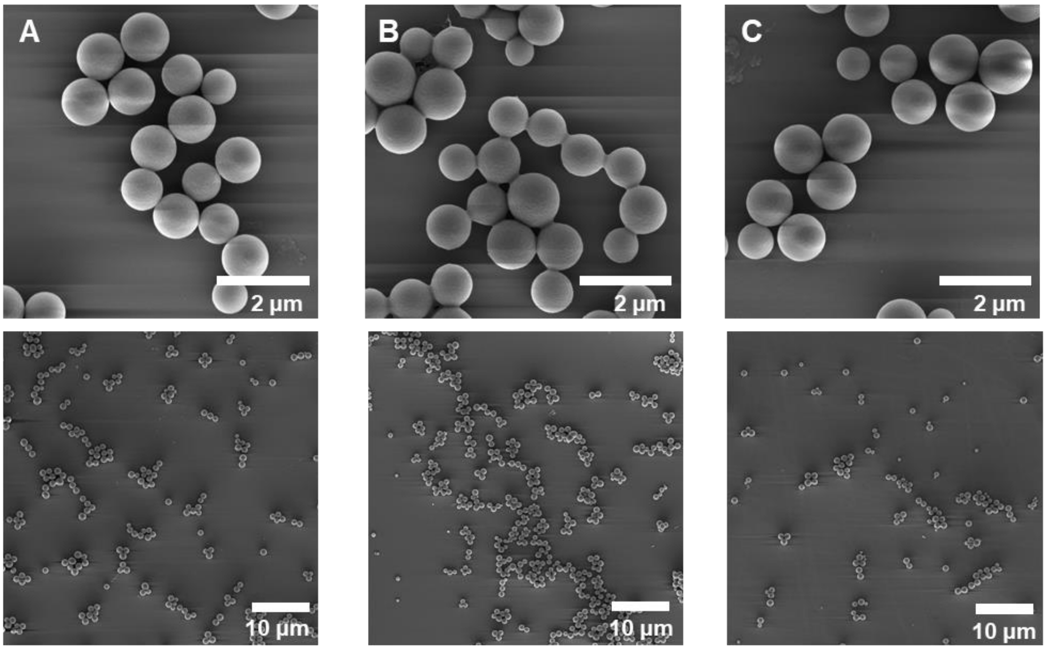

Supplementary Figure 6. Initial SEM images of AA1/BB1 particles. (run 1.1_B) redispersed in **A** ACN, **B** THF, and **C** Chloroform, time = 0.

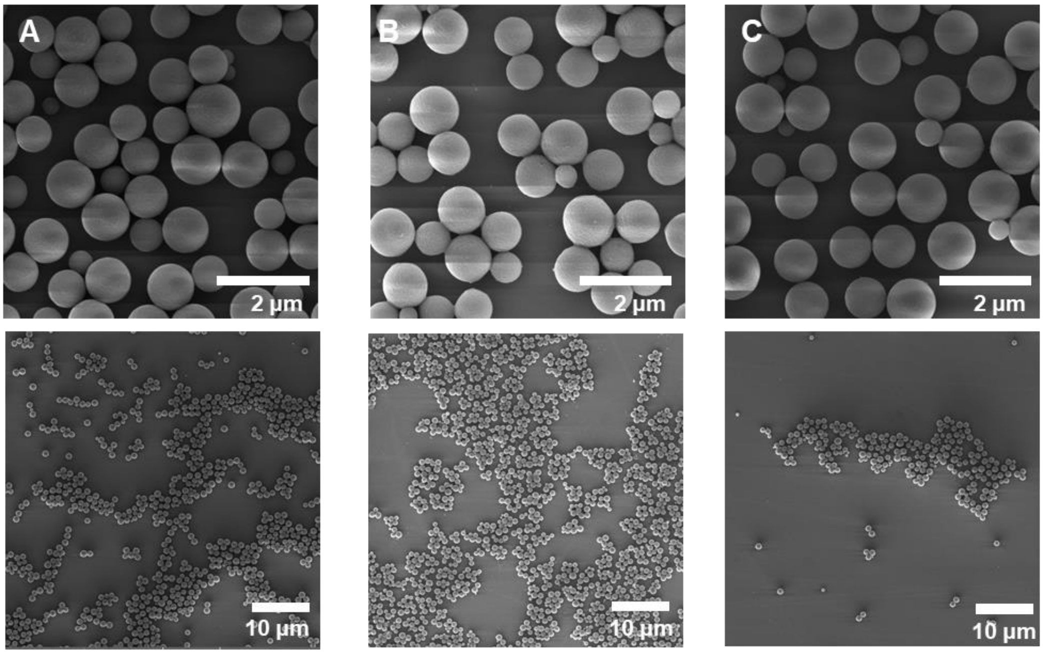

Supplementary Figure 7. SEM images of aged AA1/BB1 particles. (run 1.1_B) redispersed in **A** ACN, **B** THF, and **C** Chloroform, time = 6 months.

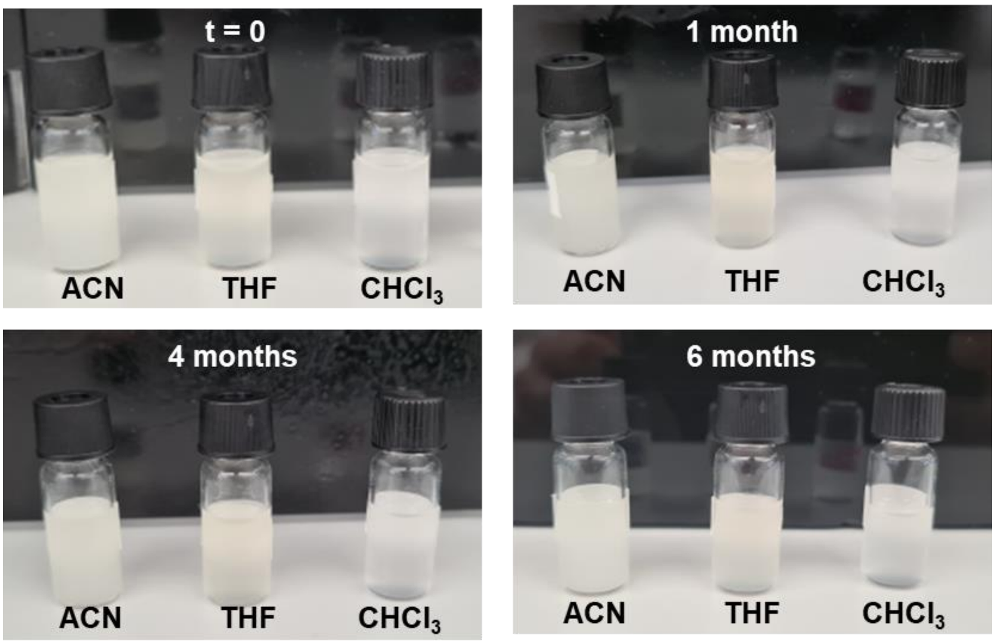

Supplementary Figure 8. Optical images of particle solution before and after aging. Pictures of the AA1/BB1 particles dispersions (run 1.1_B) in ACN, THF, and Chloroform stored at ambient temperature (25° C) after 1, 4, and 6 months. As the particles can settle, the vials were sonicated for 5 sec prior pictures and SEM.

Particles AA1/BB1 (run 1.1_D) were synthesized as per standard procedures and washed 2 times with THF. 2 mg of the isolated pellet were put aside and placed in a vial which was stored in an oven at 150 °C for 1 month. The particles were then redispersed in ACN and sonicated prior to SEM. In parallel, the isolated particles were then redispersed in 4 mL of TCB. A SEM was taken and noted as time 0. 2 mL of the solution was put in vial and stored at room temperature for 1 month while 2 mL of the solution was put in a second vial and heated at 150 °C (without stirring) in a heating block. Prior to SEM, the solution was sonicated for 30 sec to ensure a good dispersion for SEM and SEM from the same vial was taken every week.

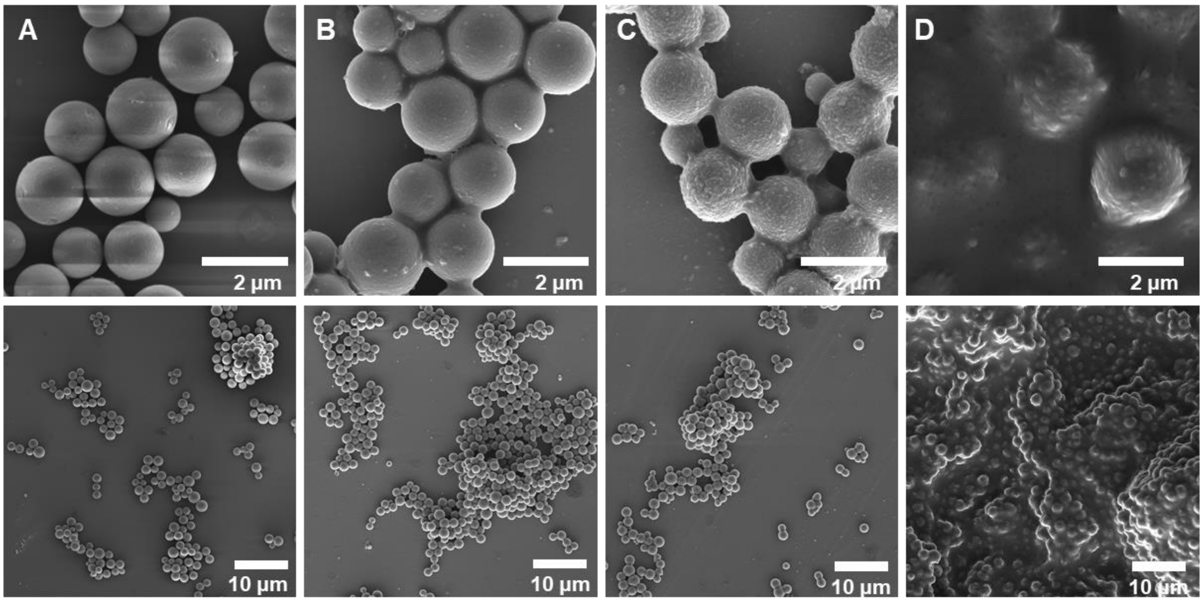

Supplementary Figure 9. **SEM pictures of the AA1/BB1 particles.** (run 1.1_D) redispersed in TCB **A** at time 0, and **B** after 1 week, **C** 2 weeks and **D** 1 month at 150 °C.

Supplementary Table 2. **Particles counting (SEM) after exposing the particles to different solvents and conditions.**

Run	Monomer AA	Monomer BB	Solvent	T (°C)	Time	D_n (µm)	*Đ*
1.1_B	1	1	ACN	25	0	1.06	1.03
1.1_B	1	1	ACN	25	6 months	1.02	1.03
1.1_B	1	1	THF	25	0	0.96	1.05
1.1_B	1	1	THF	25	6 months	0.93	1.06
1.1_B	1	1	Chloroform	25	0	0.98	1.08
1.1_B	1	1	Chloroform	25	6 months	0.91	1.12
1.1_D	1	1	ACN	25	0	1.5	1.07
1.1_D	1	1	Dry	150	1 month	1.56	1.05
1.1_D	1	1	TCB	25	0	1.55	1.08
1.1_D	1	1	TCB	25	1 month	1.53	1.09
1.1_D	1	1	TCB	150	1 week	1.65	1.06
1.1_D	1	1	TCB	150	2 weeks	1.71	1.08

6.2. DSC

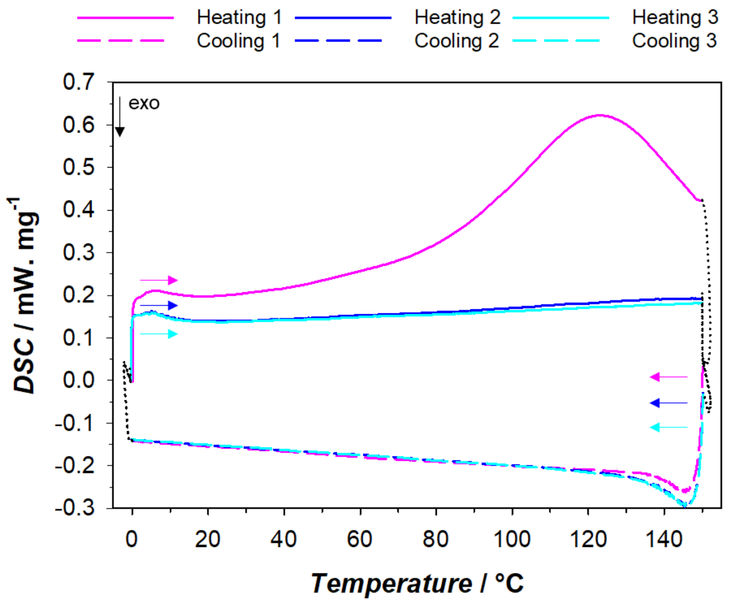

Supplementary Figure 10. DSC thermogram of AA1/BB1 particles (run 1.1_E) with 3 cycles of heating/cooling at 10 °C min^{-1} from 0 to 150 °C.

6.3. TGA

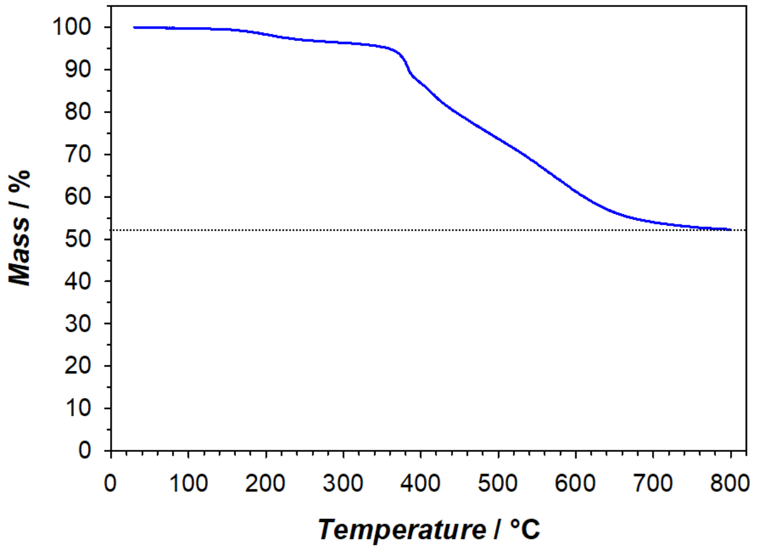

Supplementary Figure 11. **Thermogram of AA1/BB1 particles** (run 1.1_E) with a heating rate of 20 °C min^{-1} from 30-800 °C. The curve depicts the mass loss over the temperature profile.

7. Functionalization

7.1. Functionalization with tetrazole

Particles AA1/BB1 (run 1.1_E, 13.65 mg) were dispersed in 3 mL of THF as a stock solution. 4-(2-phenyl-2H-tetrazol-5-yl)benzoic acid (Tetrazole 1, 5.05 mg, 1.90 x 10^{-5} mol) and 4-(2-(4-methoxyphenyl)-2H-tetrazol-5-yl)benzoic acid (Tetrazole 2, 5.76 mg, 1.94 x 10^{-5} mol) were each dissolved in 0.5 mL of THF in a crimp vial. 1 mL of the particles stock solution was added to each solution. As blank experiment, 1 mL of the particles stock solution was also placed in a 2 mL crimp vial with 0.5 mL of THF. The dispersions were then irradiated with a UV-B light (320 nm) for 30 min. The resulting highly fluorescent dispersions were then centrifuged at 15,000 rpm for 5 min, and the sediment twice redispersed in THF followed by centrifugation to yield the isolated fluorescent, functionalised microspheres. The dry particles were redispersed in 5 mL of THF for fluorescence measurement.

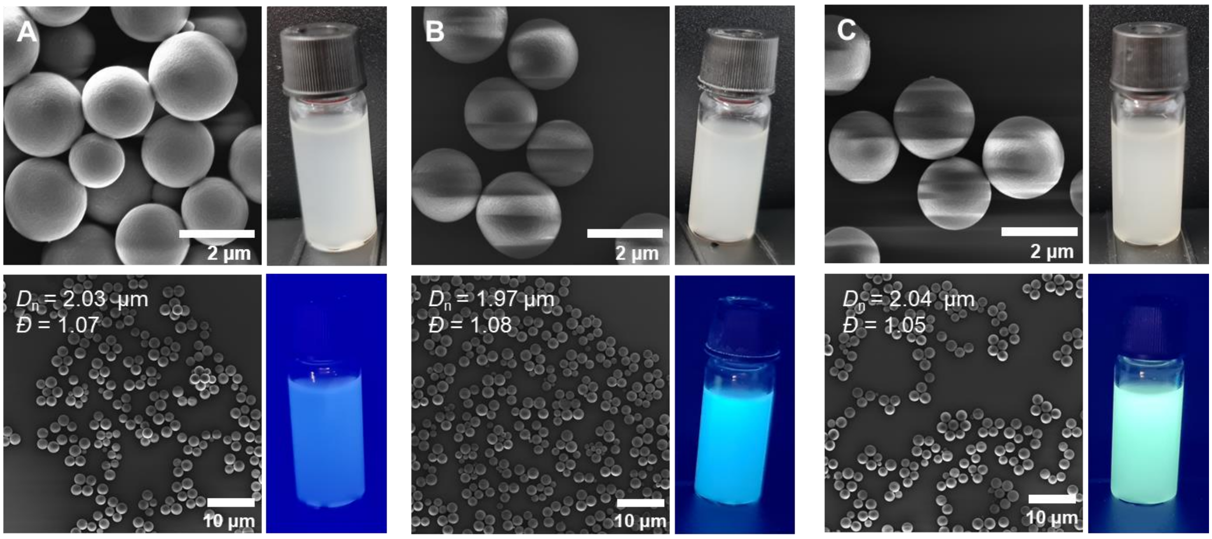

Supplementary Figure 12. **SEM pictures of the AA1/BB1 particles** (run 1.1_E) redispersed in THF and corresponding pictures of the dispersion under ambient light and under a hand-held 365 nm UV lamp. **A** Particles before functionalization, **B** After functionalization with tetrazole Tz1, and **C** after functionalization with tetrazole Tz2.

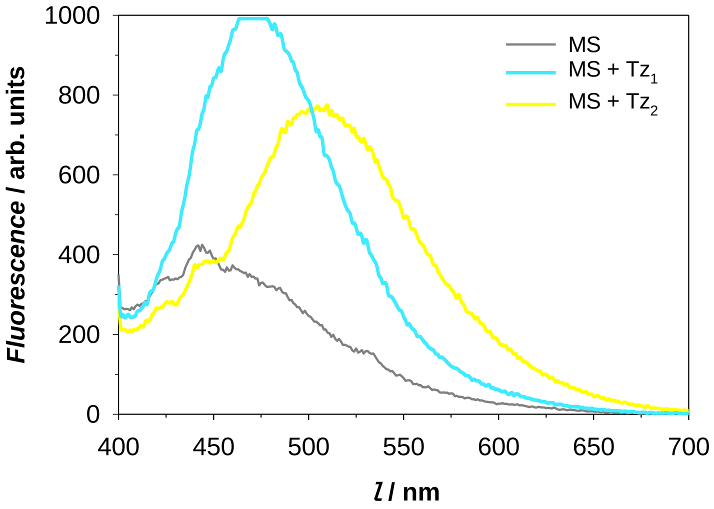

Supplementary Figure 13. **Fluorescence spectra of the AA1/BB1 particles** (run 1.1_E) redispersed in THF before functionalization and after functionalization with tetrazole 1 and 2 (λ_{ex} = 390 nm, THF, MS = Microspheres).

7.2. Functionalization with PEG-thiol

The procedure was adapted from [7].

Particles AA1/BB1 (run 1.1_F, 9.98 mg) were dispersed in 0.5 mL of chloroform and the solution was purged with Argon for 5 min in a crimp vial. In parallel, PEG-SH (2,000 g mol^{-1}, 51.41 mg, 1.2 eq.) was dissolved in 1mL of chloroform, purged for 5 min with Argon and then added to the particles dispersion under Argon. Dry triethylamine (10 µL, 3.4 eq.) was added and the reaction was stirred for 48 h on the bottle roller (10 rpm). The particles were then centrifuged at 15,000 rpm for 5 min, and the sediment twice redispersed in THF and ACN followed by centrifugation to yield the isolated functionalised microspheres. 5 mg of the particles-PEG were added to 1.5 mL of water and could easily disperse in a sonication bath in less than 10 sec. The unfunctionalized particles (5 mg) were added to 1.5 mL of water and the aggregates could not be dispersed even after 3 min in the sonication bath.

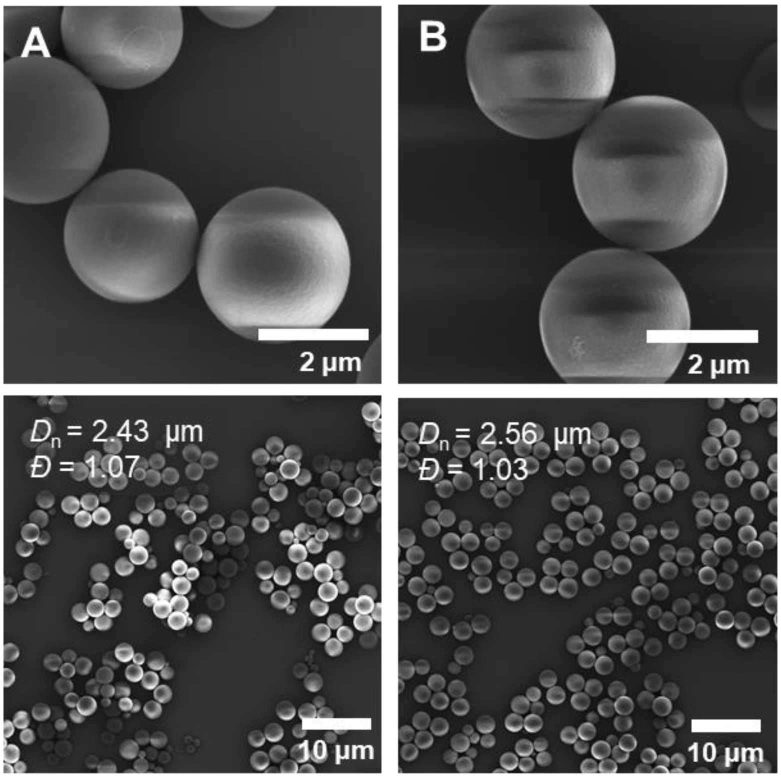

Supplementary Figure 14. **SEM pictures of the AA1/BB1 particles** (run 1.1_F) redispersed in ACN. **A** Particles before functionalization, **B** After functionalization with PEG-SH

Supplementary Table 3. **Particles counting (SEM) and reactions conditions of the particles' functionalization.** AA1/BB1 monomer (3W, 365 nm, 2 cm).

Run	Functionalization	D_n (µm)	Đ
1.1_E	None	2.03	1.07
1.1_E_Tz1	Tetrazole 1	1.97	1.08
1.1_E_Tz2	Tetrazole 2	2.04	1.05
1.1_F	None	2.43	1.07
1.1_F_PEG	PEG-SH 2K	2.56	1.03

8. NMR Spectra

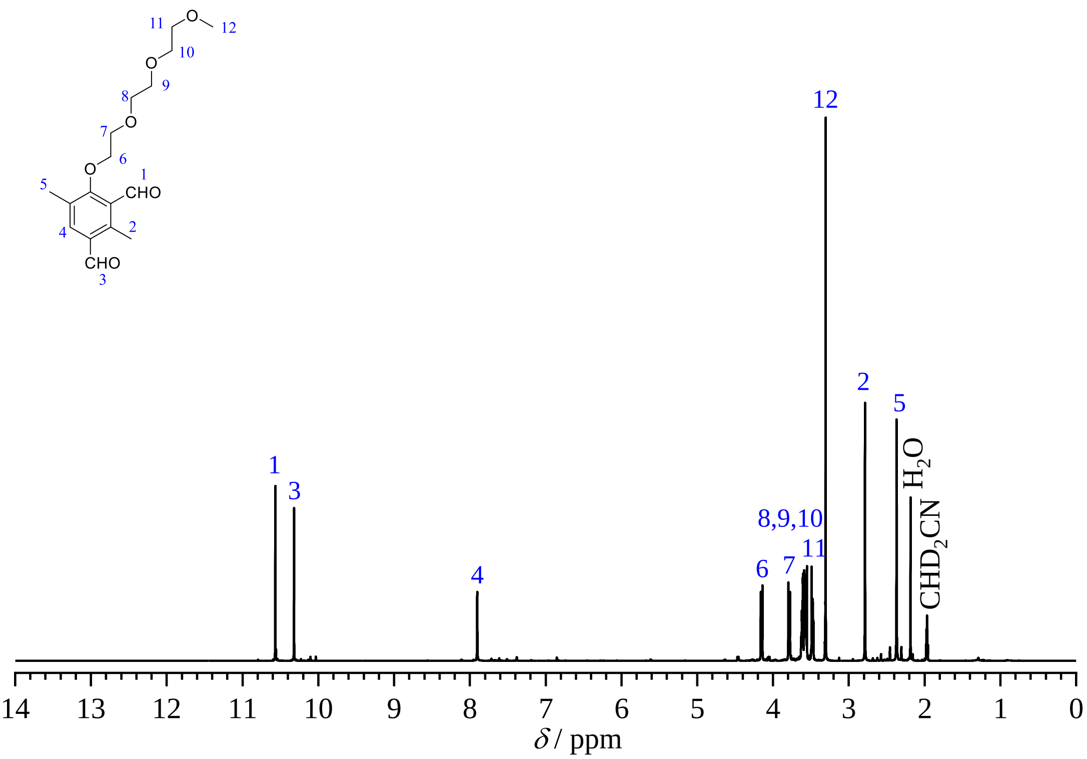

Supplementary Figure 15. **^{1}H-NMR spectrum of AA2**. (4-(2-(2-(2-methoxyethoxy)ethoxy)-ethoxy)-2,5-dimethylisophthalaldehyde) recorded in CD_3CN and assigned resonances.

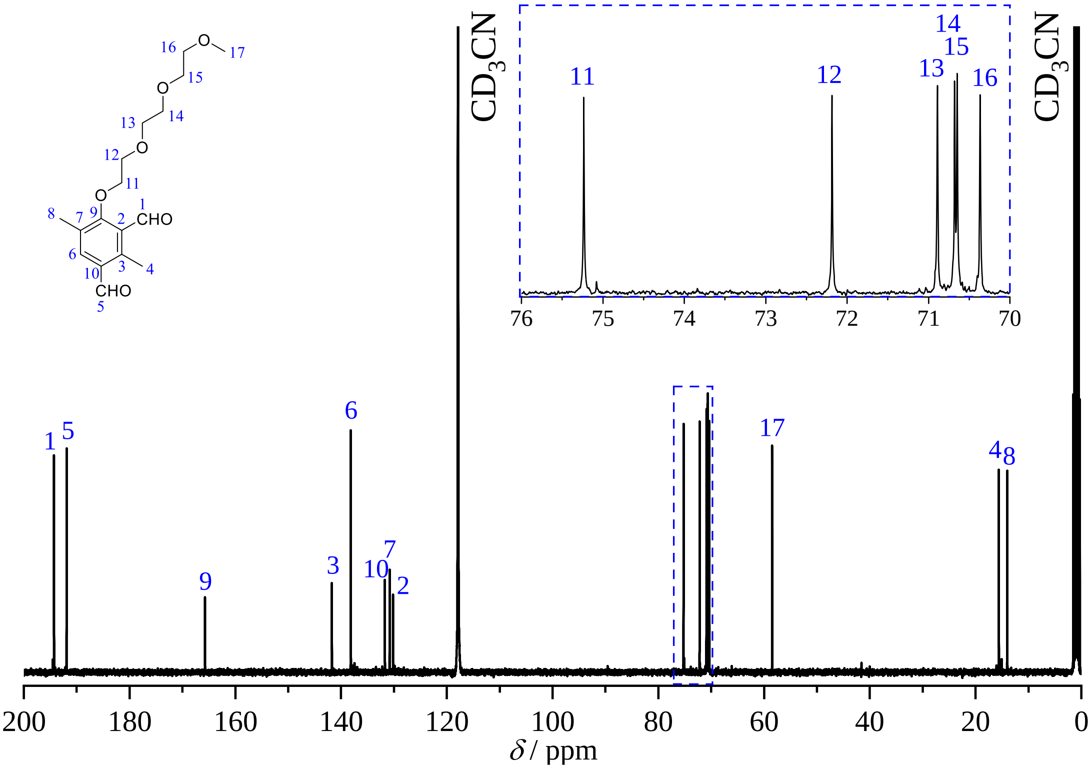

Supplementary Figure 16. ^{13}C-NMR spectrum of AA2. (4-(2-(2-(2-methoxyethoxy)ethoxy)-ethoxy)-2,5-dimethylisophthalaldehyde) recorded in CD_3CN_3 and assigned resonances.

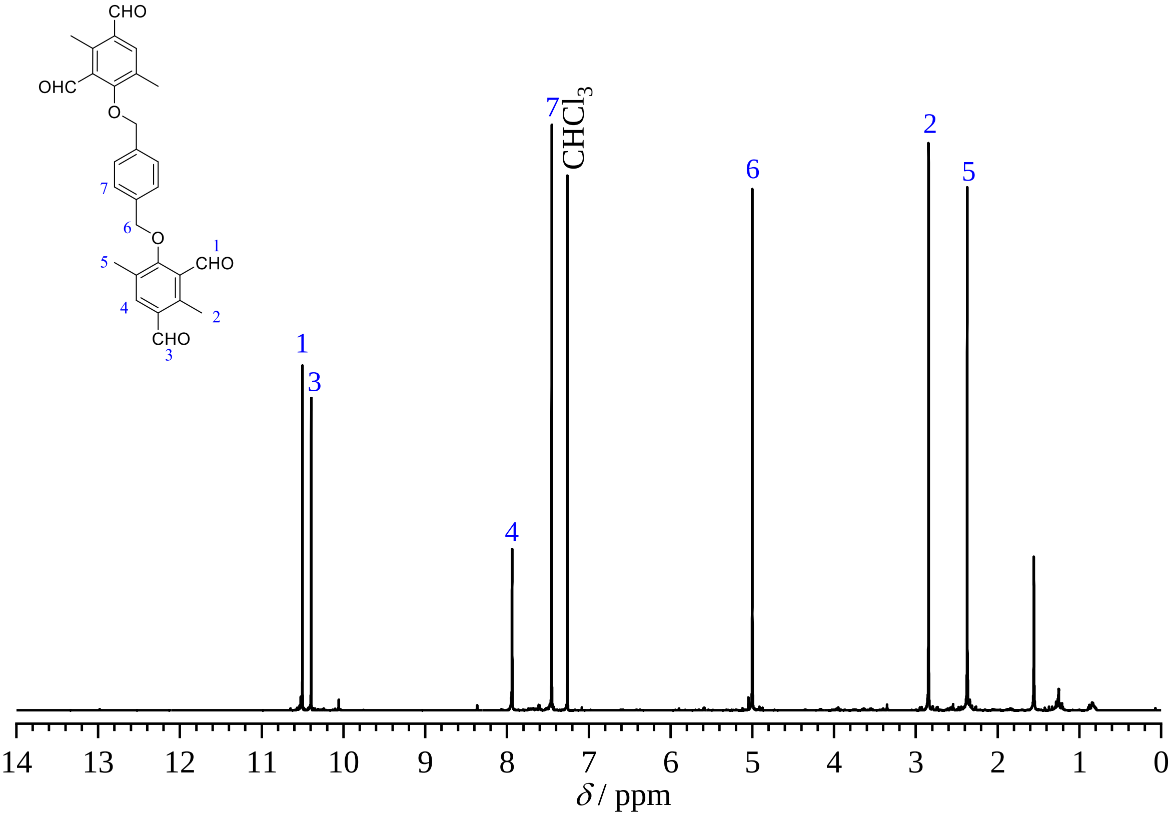

Supplementary Figure 17. **^{1}H-NMR spectrum of AA3.** (4,4'-((1,4-phenylenebis-(methylene))bis(oxy))bis(2,5-dimethylisophthalaldehyde) recorded in $CDCl_3$ and assigned resonances.

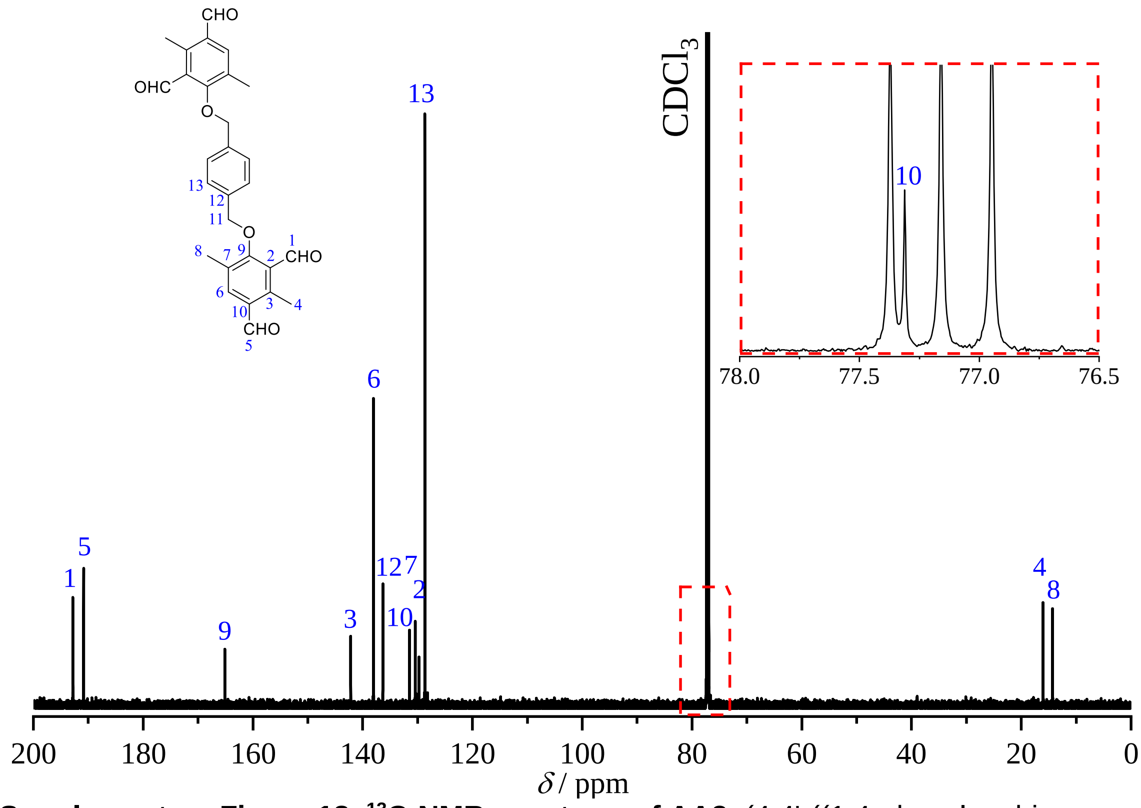

Supplementary Figure 18. **^{13}C-NMR spectrum of AA3.** (4,4'-((1,4-phenylenebis-(methylene))bis(oxy))bis(2,5-dimethylisophthalaldehyde) recorded in $CDCl_3$ and assigned resonances.

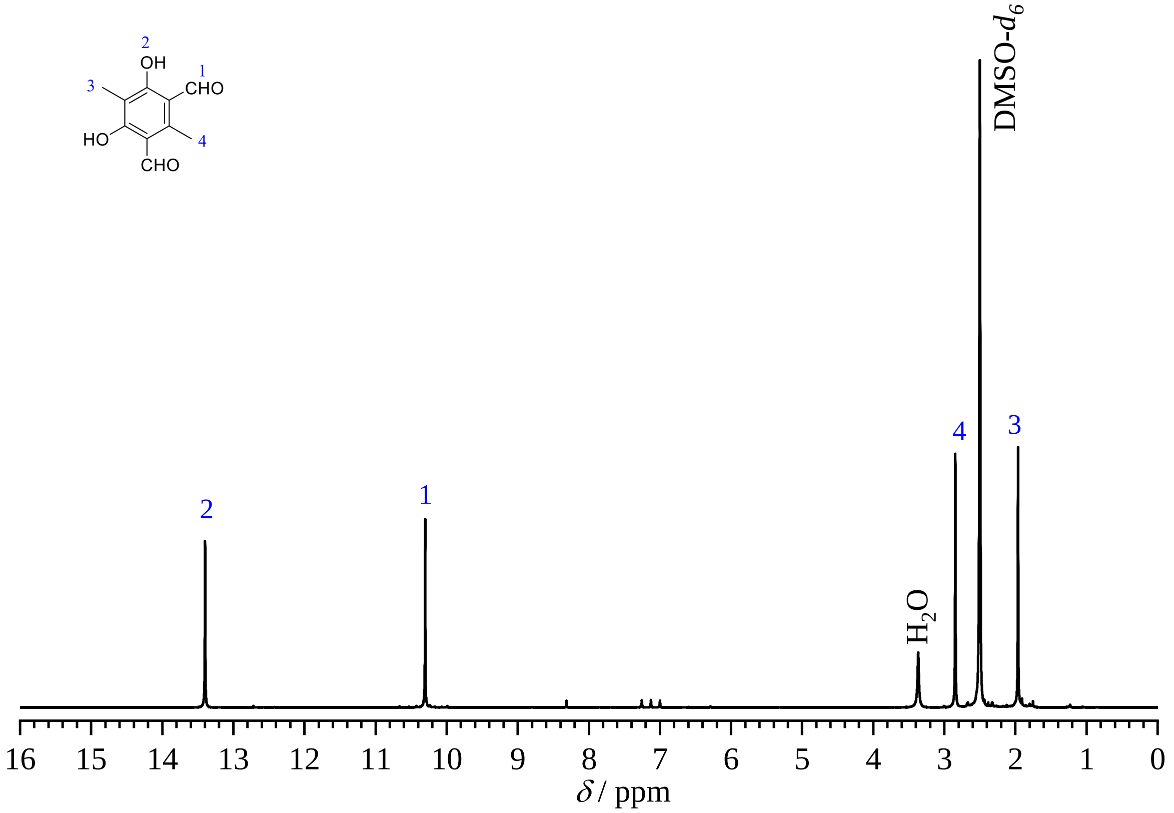

Supplementary Figure 19. **^{1}H-NMR spectrum of AA4 Step 1.** 4,6-dihydroxy-2,5-dimethyl-isophthalaldehyde recorded in DMSO-d_6 and assigned resonances.

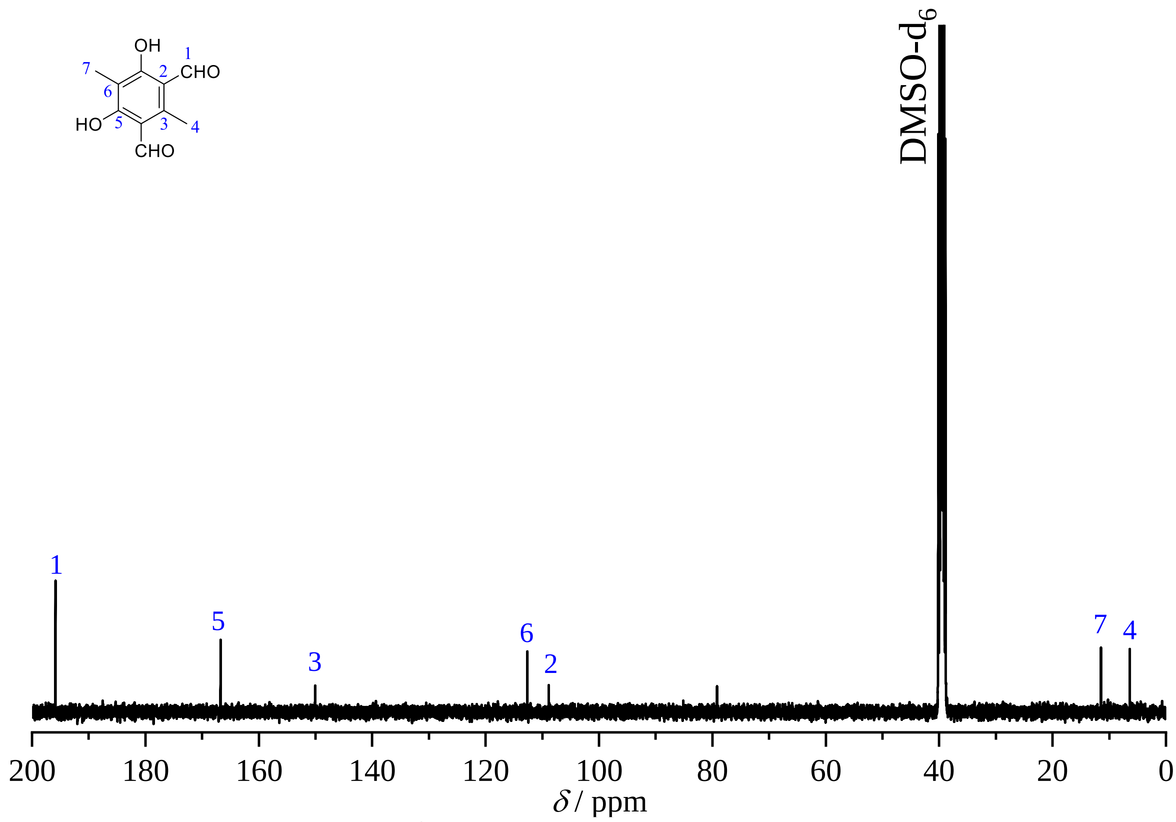

Supplementary Figure 20. ^{13}C-NMR spectrum of AA4 Step 1. 4,6-dihydroxy-2,5-dimethylisophthalaldehyde recorded in DMSO-d_6 and assigned resonances.

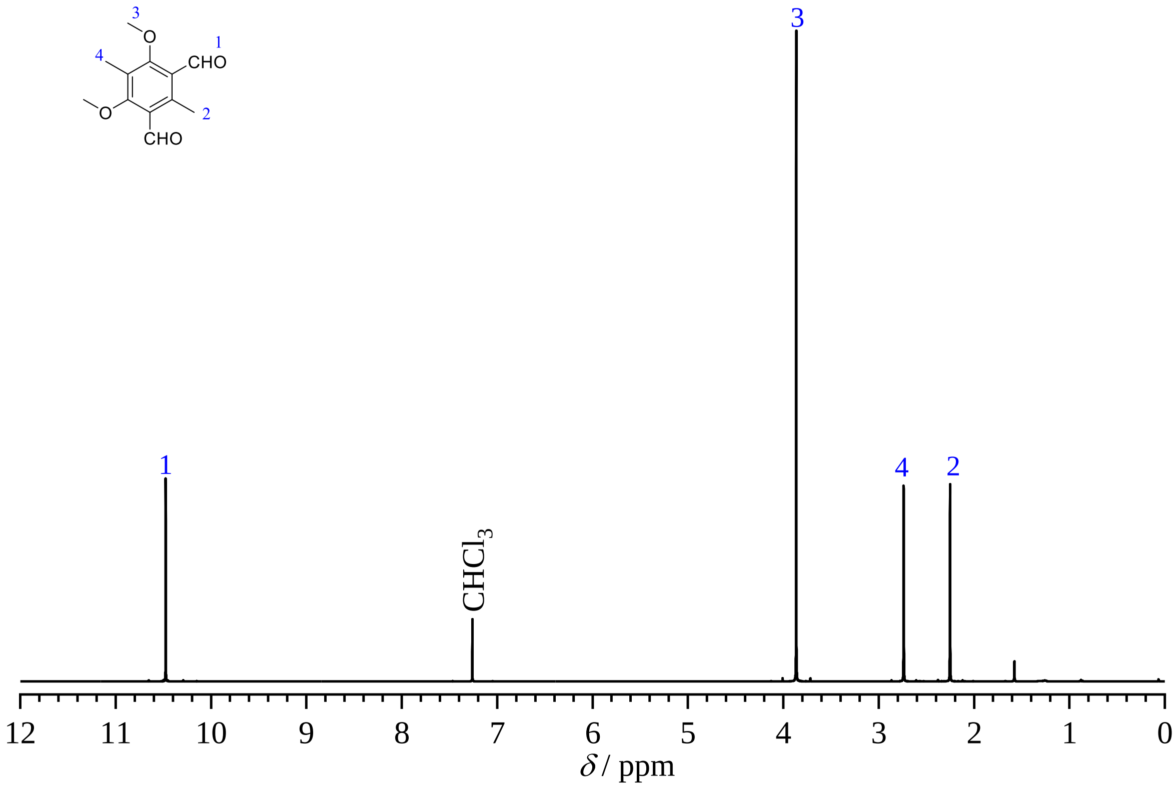

Supplementary Figure 21. **^{1}H-NMR spectrum of AA4.** 4,6-dimethoxy-2,5-dimethyliso-phthalaldehyde recorded in $CDCl_3$ and assigned resonances.

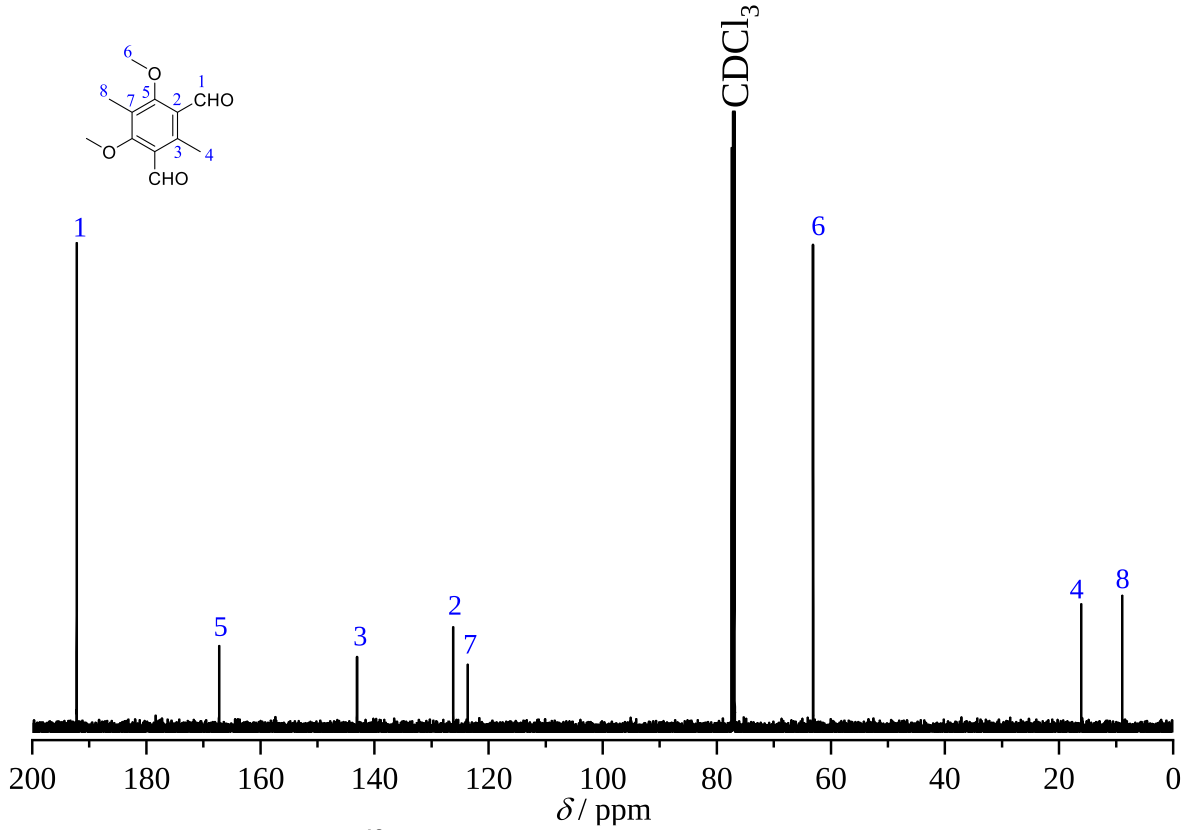

Supplementary Figure 22. **^{13}C-NMR spectrum of AA4.** 4,6-dimethoxy-2,5-dimethyliso-phthalaldehyde recorded in $CDCl_3$ and assigned resonances.

9. LCMS Results

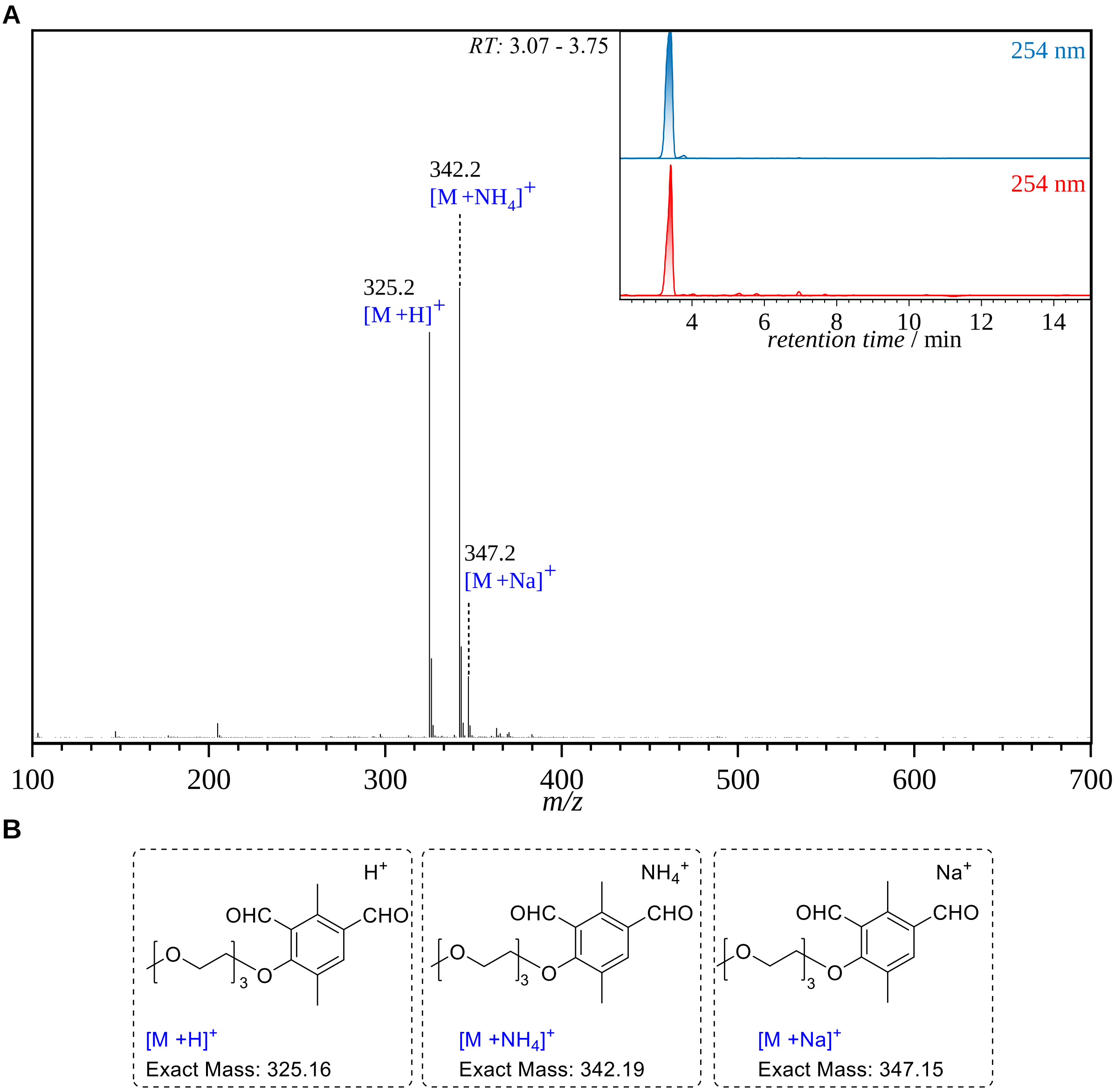

Supplementary Figure 23. LC-MS results of monomer AA2. A. LC-trace (215 and 254 nm detector wavelength) and accumulated mass-spectra of (4-(2-(2-(2-methoxyethoxy)ethoxy)-ethoxy)-2,5-dimethylisophthalaldehyde). **B.** Fragments identified in LCMS-spectrum of monomer AA2 and corresponding abbreviations

.

A

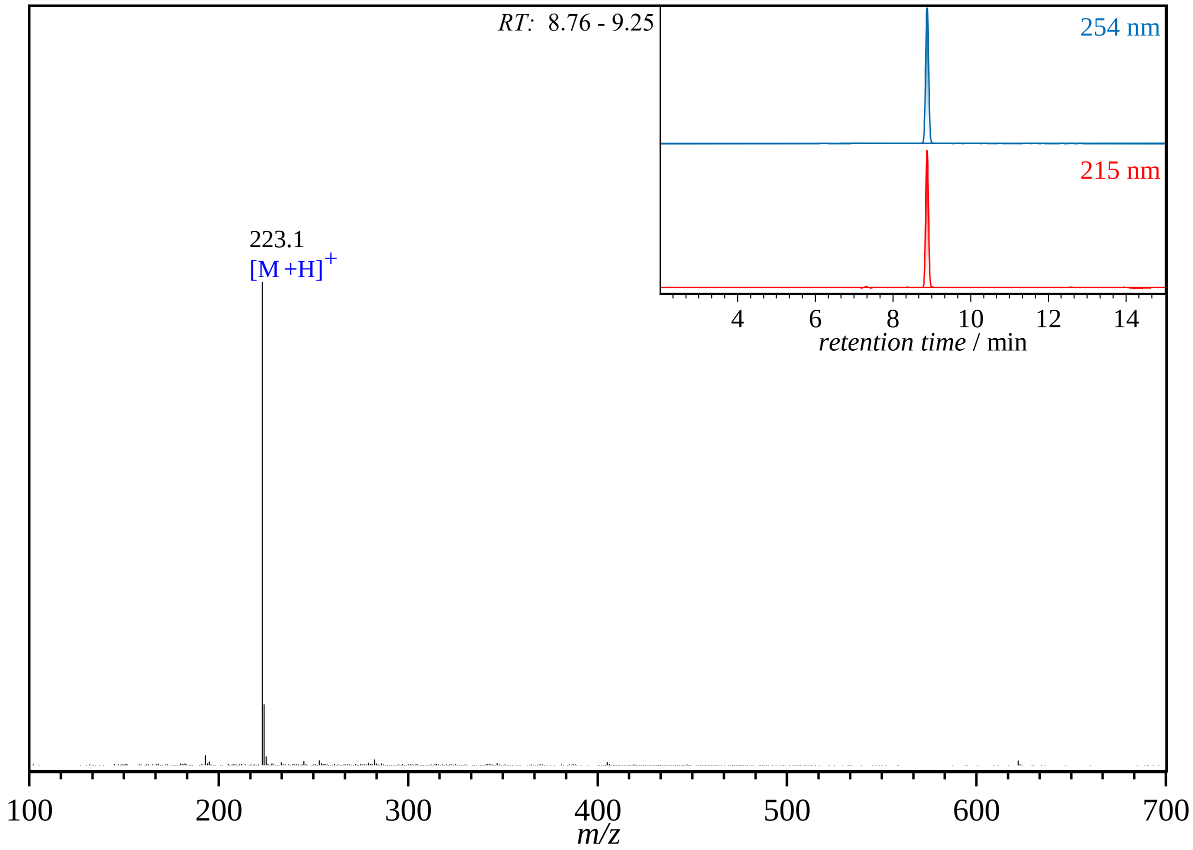

B

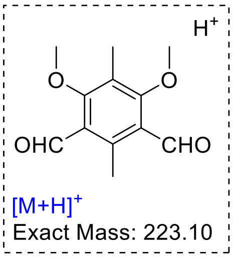

Supplementary Figure 24. LC-MS results of monomer AA4. A. LC-trace (215 and 254 nm detector wavelength) and accumulated mass-spectra and of 4,6-dimethoxy-2,5-dimethyliso-phthalaldehyde. **B.** Fragments identified in LCMS-spectrum of AA4 and corresponding abbreviations.

10. References

1. Delafresnaye, L., Schmitt, C.W., Barner, L., and Barner-Kowollik, C. (2019). A Photochemical Ligation System Enabling Solid-Phase Chemiluminescence Read-Out. Chem.: Eur. J *25*, 12538-12544. 10.1002/chem.201901858.
2. Konakahara, T., Kiran, Y., Okuno, Y., Ikeda, R., and Sakai, N. (2010). An expedient synthesis of ellipticine via Suzuki–Miyaura coupling. Tetrahedron Lett. *51*, 2335-2338. 10.1016/j.tetlet.2010.02.125
3. Feist, F., Walden, S.L., Alves, J., Kunz, S.V., Micallef, A.S., Brock, A.J., McMurtrie, J.C., Weil, T., Blinco, J.P., and Barner-Kowollik, C. (2021). Wavelength-Gated Photochemical Synthesis of Phenalene Diimides. Angew. Chem. Int. Ed. *60*, 10402-10408. https://doi.org/10.1002/anie.202016632.
4. Shopsowitz, K.E., Edwards, D., Gallant, A.J., and MacLachlan, M.J. (2009). Highly substituted Schiff base macrocycles via hexasubstituted benzene: a convenient double Duff formylation of catechol derivatives. Tetrahedron *65*, 8113-8119. https://doi.org/10.1016/j.tet.2009.07.094.
5. Bureau of Meterology, Australian Government. Daily global solar exposure.

http://www.bom.gov.au/jsp/ncc/cdio/weatherData/av?p_nccObsCode=193&p_display_type=dailyDataFile&p_startYear=&p_c=&p_stn_num=040913
6. Australian Radiation Protection and nuclear Safety Agency, https://www.arpansa.gov.au/our-services/monitoring/ultraviolet-radiation-monitoring/ultraviolet-radiation-index.
7. Discekici, E.H., St. Amant, A.H., Nguyen, S.N., Lee, I.-H., Hawker, C.J., and Read de Alaniz, J. (2018). Endo and Exo Diels–Alder Adducts: Temperature-Tunable Building Blocks for Selective Chemical Functionalization. J. Am. Chem. Soc. *140*, 5009-5013. 10.1021/jacs.8b01544.
